# *PRRT2* Mutations Are Related to Febrile Seizures in Epileptic Patients

**DOI:** 10.3390/ijms151223408

**Published:** 2014-12-16

**Authors:** Zheng-Wen He, Jian Qu, Ying Zhang, Chen-Xue Mao, Zhi-Bin Wang, Xiao-Yuan Mao, Zhi-Yong Deng, Bo-Ting Zhou, Ji-Ye Yin, Hong-Yu Long, Bo Xiao, Yu Zhang, Hong-Hao Zhou, Zhao-Qian Liu

**Affiliations:** 1 Department of Neurosurgery, the Affiliated Cancer Hospital of Xiangya School of Medicine, Central South University, Changsha 410014, China; E-Mails: hezhengw@gmail.com (Z.-W.H.); dzyong111@gmail.com (Z.-Y.D.); 2 Department of Clinical Pharmacology, Xiangya Hospital, Central South University, Changsha 410008, China; E-Mails: seemore126@gmail.com (J.Q.); yz_fly@csu.edu.cn (Y.Z.); sharemix@csu.edu.cn (C.-X.M.); wangzhibinup@gmail.com (Z.-B.W.); xiaoyuanm@csu.edu.cn (X.-Y.M.); yinjiye@csu.edu.cn (J.-Y.Y.); zbtinggggg@gmail.com (Y.Z.); honghaozhou2012@gmail.com (H.-H.Z.); 3 Institute of Clinical Pharmacology, Central South University, Hunan Key Laboratory of Pharmacogenetics, Changsha 410078, China; E-Mail: botingzhou0918@gmail.com; 4 Departments of Pharmacy and Neurology, Xiangya Hospital, Central South University, Changsha 410008, China; E-Mails: longhongyu@csu.edu.cn (H.-Y.L.); boxiaoxiao123@gmail.com (B.X.)

**Keywords:** proline-rich transmembrane protein 2, febrile seizures, mutation, epilepsy

## Abstract

Previous studies reported that the proline-rich transmembrane protein 2 (*PRRT2*) gene was identified to be related to paroxysmal kinesigenic dyskinesia (PKD), infantile convulsions with PKD, PKD with migraine and benign familial infantile epilepsy (BFIE). The present study explores whether the *PRRT2* mutation is a potential cause of febrile seizures, including febrile seizures plus (FS+), generalized epilepsy with febrile seizures plus (GEFS+) and Dravet syndrome (DS); thus, it may provide a new drug target for personalized medicine for febrile seizure patients. We screened *PRRT2* exons in a cohort of 136 epileptic patients with febrile seizures, including FS+, GEFS+ and DS. *PRRT2* genetic mutations were identified in 25 out of 136 (18.4%) febrile seizures in epileptic patients. Five loss-of-function and coding missense mutations were identified: c.649delC (p.R217Efs*12), c.649_650insC (p.R217Pfs*8), c.412C>G (p.Pro138Ala), c.439G>C (p.Asp147His) and c.623C>A (p.Ser208Tyr). *PRRT2* variants were probably involved in the etiology of febrile seizures in epileptic patients.

## 1. Introduction

There is a long-known relationship between human epilepsies and other paroxysmal brain disorders, such as paroxysmal dyskinesia, episodic ataxia or migraine. They are all involuntary movements, which are spontaneous or triggered by various types of stimuli [1]. Several previous research studies described proline-rich transmembrane protein 2 (*PRRT2*) as the causative gene in some nervous system diseases, including paroxysmal kinesigenic dyskinesia (PKD), infantile convulsions with PKD, benign familial infantile epilepsy (BFIE), hemiplegic migraine and episodic ataxia alone [1,2,3,4,5,6,7,8,9,10,11,12,13]. Evidence for shared pathophysiologic mechanisms underlying the concurrence of benign infantile convulsions (IC) and of PKD in the same patients or families was obtained [8,13]. *PRRT2* gene consists of four exons, encoding proline-rich transmembrane protein 2, and maps to chromosome 16p11.2, one of the known BFIE loci [2,14]. The PRRT2 protein is highly expressed in the developing nervous system and localized in the axons [6]. Although its interaction with synaptosomal-associated protein 25 (SNAP25) suggests important roles in synaptic vesicle docking and exocytosis, the function of the *PRRT2* gene is poorly understood [2,14,15]. Most of the pathological mutations in the *PRRT2* gene discovered cause truncation of the protein, leading to loss of function. Among mutations in *PRRT2*, c.649dupC has been shown to be a mutation hotspot recently [9,14,15,16,17,18,19,20,21,22]. Some studies on *PRRT2* mutations in a small number of atypical benign familial and infantile epilepsies [9,14,15] provided evidence of the association between febrile seizures or childhood absence seizures and *PRRT2* mutations; however, the presence of *PRRT2* mutations in a broader spectrum of epileptic phenotypes has not been investigated clearly.

In this study, we aim to widen the spectrum of phenotypes associated with *PRRT2* mutations and investigate whether *PRRT2* mutations are involved in febrile seizure-related epilepsy, including febrile seizures plus (FS+), generalized epilepsy with febrile seizures plus (GEFS+) and Dravet syndrome (DS).

## 2. Results and Discussion

**Figure 1:**
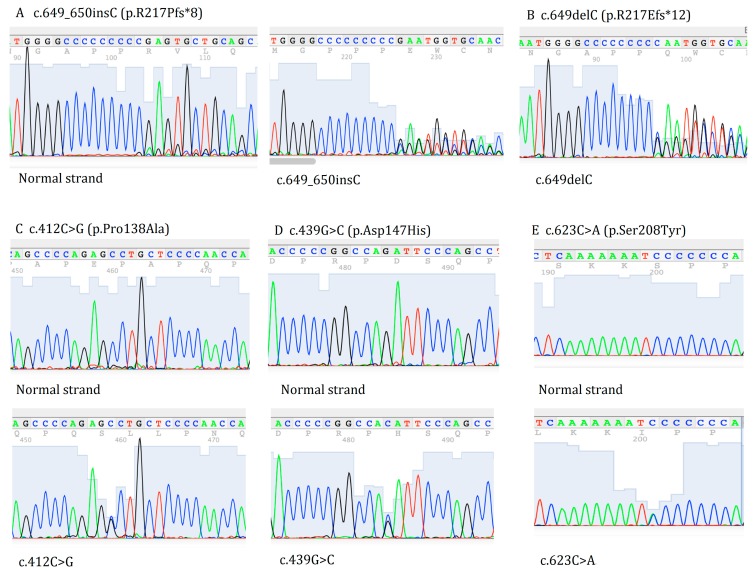
The identified proline-rich transmembrane protein 2 (*PRRT2*) mutations in epileptic patients with febrile seizures. (**A**) c.649_650insC (p.R217Pfs*8); (**B**) c.649delC (p.R217Efs*12); (**C**) c.412C>G (p.Pro138Ala); (**D**) c.439G>C (p.Asp147His) and (**E**) c.623C>A (p.Ser208Tyr).

Two truncating frameshift mutations and three missense mutations in 136 patients with febrile seizure-related epilepsy were identified by sequencing of the *PRRT2* gene (Figure 1). Among these mutations, p.Arg217Glufs*12 (c.649delC) was found in two patients (one FS+, one DS); p.R217Pfs*8 (c.640_641insC) was found in four patients (two FS+, one GEFS+, one DS); c.412C>G (p.Pro138Ala) was found in 14 patients (eight FS+, two GEFS+, four DS); c.439G>C (p.Asp147His) was found in five patients (four GEFS+, one DS); and c.623C>A (p.Ser208Tyr) was found in one DS patient. None of these mutations were found in 108 healthy Chinese controls. The characteristics of 25 mutated patients are shown in Table 1.

**Table 1:** Characteristics of *PRRT2* mutations in epileptic patients with febrile seizures. M, male; F, female; d, days; w, weeks; m, months; y, years; FS+, febrile seizures plus; GEFS+, generalized epilepsy with febrile seizures plus; DS, Dravet syndrome; N, normal; ND, not done; AB, abnormal; CBZ, carbamazepine; PHT, phenytoin; OXC, oxcarbazepine; VPA, sodium valproate; LEV, levetiracetam; TPM, topiramate; PB, phenobarbitone. All patients had heterozygous mutations.

Case Number	Gender	Age (year)	Age at Onset	Subtypes	Familial/Sporadic	GGE/MRI/CT	Duration of Seizure	Frequency of Seizure	Current Medication	Nucleotide Changes	Amino Acid Changes
89	M	7	2 y	FS+	Sporadic	N/N/N	<20 s	1–2 m	CBZ	c.412C>G	p.Pro138Ala
123	F	18	13 y	FS+	Sporadic	N/N/ND	5–10 s	1–2 m	VPA	c.623C>A	p.Ser208Tyr
174	M	16	2 y	FS+	Sporadic	AB/N/N	<30 s	4–5 m	CBZ, PB	c.412C>G	p.Pro138Ala
114	M	6	2 y	FS+	Sporadic	N/ND/N	1 min	10–15 y	CBZ	c.412C>G	p.Pro138Ala
192	M	13	1 y	FS+	Sporadic	N/AB/N	10 s	2–3 y	VPA, LTG	c.439G>C	p.Asp147His
311	M	6	4 y	FS+	Sporadic	AB/N/N	3–5 min	3–5 m	LEV	c.412C>G	p.Pro138Ala
313	M	6	3 y	FS+	Sporadic	N/N/N	10 s	2–4 m	VPA, PHT	c.412C>G	p.Pro138Ala
SN540	M	18	5 m	DS	Familial	AB/ND/ND	1–2 min	1–2 m	CBZ, OXC	c.640_641insC	p.R217Pfs*8
363	M	33	9 y	FS+	Sporadic	AB/N/N	3–10 s	1 m	CBZ,PHT	c.640_641insC	p.R217Pfs*8
										c.439G>C	p.Asp147His
737	M	7	4 y	FS+	Sporadic	N/ND/N	2–5 s	2 m	VPA	c.412C>G	p.Pro138Ala
853	M	17	8 y	FS+	Sporadic	N/ND/ND	2–5 min	4–8 y	CBZ	c.412C>G	p.Pro138Ala
HH69	M	15	3 y	FS+	Sporadic	N/N/AB	1 min	1–2 m	VPA	c.439G>C	p.Asp147His
SN252	M	24	4 y	FS+	Sporadic	AB/N/N	2 min	2–3 y	CBZ, VPA	c.439G>C	p.Asp147His
SN488	F	20	13 y	FS+	Sporadic	AB/N/N	2–3 min	2–3 m	VPA, TPM	c.412C>G	p.Pro138Ala
SN275	F	12	3 y	FS+	Sporadic	AB/N/N	2–4 min	1–2 y	CBZ	c.649delC	p.Arg217Glufs*12
812	M	32	10 y	GEFS+	Familial	N/ND/ND	5–6 min	4–6 d	CBZ	c.412C>G	p.Pro138Ala
1232	M	15	2 y	GEFS+	Familial	N/ND/N	1–3 min	2 w	VPA	c.412C>G	p.Pro138Ala
SN854	M	36	8 y	GEFS+	Familial	N/ND/ND	1–2 min	2 d	CBZ, PB	c.640_641insC	p.R217Pfs*8
576	F	5	5 m	DS	Sporadic	N/ND/N	1 min	10 w	CBZ, VPA	c.439G>C	p.Asp147His
1186	F	10	8 m	DS	Sporadic	N/N/ND	1–3 min	2–3 w	CBZ, VPA	c.412C>G	p.Pro138Ala
872	F	3	9 m	DS	Sporadic	AB/N/N	10 min	1 w	CBZ, LEV	c.412C>G	p.Pro138Ala
SN676	M	32	5 m	DS	Sporadic	N/ND/ND	2–3 min	2–3 d	OXC, VPA	c.649delC	p.Arg217Glufs*12
SN740	F	25	40 d	DS	Sporadic	AB/N/N	1–2 min	3 m	CBZ, VPA	c.412C>G	p.Pro138Ala
SN540	M	18	5 m	DS	Familial	AB/N/N	1–2 min	1–2 m	CBZ, OXC	c.640_641insC	p.R217Pfs*8
428	M	12	11 m	DS	Sporadic	AB/ND/N	1 min	1–2 w	LEV, VPA	c.412C>G	p.Pro138Ala

p.R217Pfs*8 (c.640_641insC) was the common mutation in benign familial infantile epilepsy (BFIE), infantile convulsions and choreoathetosis (ICCA) and PKD [1,2,4,8,9,14,23]. We also found that two FS+ patients, one GEFS+ patient and one DS patient have this mutation. p.Arg217Glufs*12 (c.649delC) was found in two patients. Moreover, three missense mutations were found in the dbSNP database and were designated as rs79182085 (c.412C>G), rs79568162 (c.439G>C) and rs201409113 (c.623C>A). SIFT [24] predicted the first two variants to be “tolerated” and the last variant as “damaging”. PholyPhen [25] classified the first two variants as “benign” and the last variant as “probably damaging”. However, the performance of these prediction programs on the wild-type residues of variation sites is known to be low [26]. The allelic frequencies of these three SNPs in our cohort were 0.051, 0.018 and 0.0036, respectively. Rs79182085 allelic frequency is 0.158 in dbSNP in the Chinese and Japanese population. Frequency data for the other two SNPs are not available in dbSNP. Moreover, these five mutations were not found in our healthy controls.

PRRT2 is a transmembrane protein that is capable of binding to SNAP25 [2]. Most of the pathological mutations in the *PRRT2* gene that have been discovered to date cause truncation of the protein, leading to loss of function [14,27]. A previous study has elucidated the role of *PRRT2* mutations in BFIE and provided evidence of associations with febrile seizures and childhood absence seizures [4]. Ingrid Scheffer and colleagues screened out eight individuals with *PRRT2* mutations exhibiting febrile seizures or febrile seizures plus [9]. However, some studies found no mutations in epileptic encephalopathies and benign and severe infantile seizures [16,28]. In the current study, we found some truncating frameshift mutations and missense mutations presenting in the febrile seizure-related epileptic patients. Although the mutations were not as common as in PKD and BFIE patients, especially the hotspot mutation c.649_650insC (p.P217fsX7), this still suggests that the *PRRT2* gene mutant in febrile seizures is related to epilepsy, including FS+, GEFS+ and DS.

## 3. Experimental Section

### 3.1. Subjects

One hundred thirty six patients with fever-related epilepsy (74 FS+, 34 GEFS+ and 28 DS) and 108 matched healthy controls from Xiangya hospital and the Second Xiangya Hospital of Central South University were recruited in this study. The healthy controls were randomly selected from the people coming to the hospital for physical examination without any illness, and they were matched with patient groups. The diagnostic criteria were defined according to the international classification [29,30]. Informed consent was obtained from all patients or their parents to participate in the study. The Ethics Committee of Xiangya School of Medicine and the Ethics Committee of Institute of Clinical Pharmacology of Central South University approved the study. The clinical study admission (Registration Number: ChiCTR-TCH-0000813, date of approval: 29 April 2010 04/29/2010) was approved by the Chinese Clinical Trial Register. All patients consented to their data being used for research.

### 3.2. Mutation Analysis of the Proline-Rich Transmembrane Protein 2 (PRRT2) Gene

DNA was extracted from whole blood according to previous standard procedures [31]. All *PRRT2* gene coding exons were screened for mutations by PCR amplification and Sanger sequencing. Primers for mutational analysis of all *PRRT2* gene coding exons were followed as previously described (Table 2) [23].

**Table 2:** Primer sequences of the *PRRT2* gene. *Exon2A*: *PRRT2* gene exon 2 fragment A; *Exon2B: PRRT2* gene exon 2 fragment B; *Exon3–4*: *PRRT2* gene exon 3 and exon 4.

*PRRT2*	Forward Primer	Reverse Primer
*Exon2A*	5'-ctcctcctcttccagggttt-3'	5'-tttttgagggtggtgagtga-3'
*Exon2B*	5'-tctgagagtgtaggggaaaagc-3'	5'-ctagggagaggcaaacaaagg-3'
*Exon3–4*	5'-tccacctgatcccttctgg-3'	5'-caggctcccttggtccttag-3'

## 4. Conclusions

Nowadays, although more and more research has been focused on the susceptibility and drug efficacy of epilepsy, there are still some patients who cannot benefit from the anti-epileptic drug efficacy. The susceptibility genes as drug targets play a key role in the optimization of treatment. As a new susceptibility gene of epileptic patients with febrile seizures, mutations of *PRRT2* might underlie a broader range of seizure subtypes than what was previously suspected. Overall, *PRRT2* mutations were probably involved in the etiology of febrile seizure-related epilepsy. As a new susceptibility gene, the PRRT2 gene may provide a new drug target for personalized medicine for febrile seizure patients.
